# Cannula and circuit management in peripheral extracorporeal membrane oxygenation: An international survey of 45 countries

**DOI:** 10.1371/journal.pone.0227248

**Published:** 2019-12-30

**Authors:** Taressa Bull, Amanda Corley, India Lye, Amy J. Spooner, John F. Fraser

**Affiliations:** 1 Critical Care Research Group, The Prince Charles Hospital and University of Queensland, Chermside, Queensland, Australia; 2 Adult Intensive Care Services, The Prince Charles Hospital, Chermside, Queensland, Australia; Azienda Ospedaliero Universitaria Careggi, ITALY

## Abstract

Effective and safe practices during extracorporeal membrane oxygenation (ECMO) including infection precautions and securement of lines (cannulas and circuits) are critical to prevent life-threatening patient complications, yet little is known about the practices of bedside clinicians and data to support best practice is lacking. Therefore, the aim of this study was to identify and describe common line-related practices for patients supported by peripheral ECMO worldwide and to highlight any gaps for further investigation. An electronic survey was conducted to examine common line practices for patients managed on peripheral ECMO. Responses were obtained from 45 countries with the majority from the United States (n = 181) and United Kingdom (n = 32). Standardised infection precautions including hand hygiene, maximal barrier precautions and skin antisepsis were commonplace for cannulation. The most common antisepsis strategies included alcohol-based chlorhexidine gluconate (CHG) for cannula insertion (53%) and maintenance (54%), isopropyl alcohol on circuit access ports (39%), and CHG-impregnated dressings to cover insertion sites (36%). Adverse patient events due to line malposition or dislodgement were reported by 34% of respondents with most attributable to ineffective securement. Centres ‘always’ suturing peripheral cannula sites were more likely to experience a cannula adverse event than centres that ‘never’ sutured (35% [95% CI 30, 41] vs 0% [95% CI 0, 28]; Chi-square 4.40; *p* = 0.04) but this did not meet the *a priori* significance level of <0.01. An evidence-based guideline would be beneficial to improve ECMO line management according to 78% of respondents. Evidence gaps were identified for antiseptic agents, dressing products and regimens, securement methods, and needleless valves. Future research addressing these areas may provide opportunities for consensus guideline development and practice improvement.

## Introduction

Extracorporeal membrane oxygenation (ECMO) is an invasive method of cardiopulmonary support increasingly used in intensive care units worldwide for patients with respiratory and/or cardiac failure[1] and is high-risk for complications. The use of large-bore cannulas and associated circuit tubing (‘ECMO lines’) permit vascular access and blood flow through the extracorporeal system. Peripheral cannulations are commonly employed as they allow rapid bedside access to ECMO support using percutaneous methods[2]. The success of ECMO is dependent on effective placement and securement of these lines, among other factors. Ineffective securement can contribute to blood flow impairments, compromising patient haemodynamics and oxygenation, or cannula dislodgement which can result in air embolism or catastrophic bleeding[3]. At worst, fatal outcomes occur[4, 5].

Nosocomial infections, which include ECMO cannula-related infections (CRI), are among the most frequent complications in ECMO and cause patient morbidity[6–10] and mortality[11–13], as well as mechanical circuit dysfunction[14] which adds to patient complications. CRIs are often under-recognised contributors to infectious complications during ECMO[15]. In the limited number of studies reporting ECMO CRIs specifically, these infections account for 10% to 18% of infectious episodes[1, 16–18] and have an incidence of up to 17.2 per 1000 ECMO days[1], suggesting that they are of significant concern. Moreover, if the patient has a primary bloodstream infection (BSI) the ECMO cannula should be implicated as the culprit if the causative pathogen cannot be located in another organ, according to Messika et al.[15]. Unlike other invasive lines that undergo routine replacement for suspected or proven infections, ECMO CRIs pose a significant clinical dilemma due to the difficult and hazardous nature of line replacement[19, 20].

Critically, prevention of many of the possible complications in ECMO relies on effective practices to maintain well secured, infection-free lines, yet data to guide clinicians is lacking. Data limited to the United States shows variability in cannula insertion and maintenance practices among ECMO centres[21]. Available recommendations[20, 22] are based largely on evidence only partially applicable to ECMO lines and there is insufficient high-quality evidence specifically to guide effective line securement, dressing and access practices as a means to prevent dislodgement and/or infection in ECMO.

A better understanding of contemporary bedside practices may contribute towards the development of quality data to guide management of ECMO patients. Our aim was to identify and describe common line-related practices for patients supported by peripheral ECMO worldwide and to highlight any gaps for further investigation.

## Materials and methods

A cross-sectional, descriptive survey was conducted over a one-month period using an online survey platform (SurveyMonkey Inc., San Mateo, C.A., USA). Approval to conduct the study as an audit exempt from full ethical review was obtained by the relevant hospital human research ethics committee (HREC/15/QPCH/49). The study was endorsed by the International ECMO Network (ECMONet). Directors and coordinators from 396 ECMO centres in 51 countries that were members of the Extracorporeal Life Support Organization (ELSO) were invited by email to participate using an electronic survey link. These positions were specifically targeted as they would most likely be aware of local policy and practice. The invitation included instructions requesting only one director or coordinator complete the survey from their respective centre and to select the responses which best described that centre’s standard practices. A reminder email was sent two weeks after survey activation then resent one week before the survey closed. Participation was voluntary and anonymous, and submission verified consent to participate.

Data were collected through a 30-item investigator-developed questionnaire covering questions on centre characteristics and cannulation methods, peripheral lines (cannula insertion, dressings and securement) and circuit access, and clinical practice guidelines (refer to S1 Appendix). The questionnaire tool was assessed by an expert panel of multidisciplinary ECMO clinicians from our centre including doctors, nurses and perfusionists for feasibility, readability, understandability, ease of response, and content validity. The tool was pilot tested at two different time points by 10 ECMO clinicians to examine test-retest reliability and internal consistency. Inter-rater agreement was good with an average proportion of agreement of 0.83 across questions and average Gwet's agreement coefficient[23] of 0.73. Inter-rater agreement analyses were performed using the ‘KAPPAETC’ module[24] in the Stata Statistical Software package (StataCorp LP, version 13, College Station, Texas, USA).

Survey data were collected anonymously and downloaded into a statistical software package (SPSS Statistics for Windows, version 24, IBM Corp, Armonk, N.Y., USA) for analysis using descriptive and inferential statistics. Frequencies and percentages were calculated for the responses to each question. To test the null hypothesis of no difference in proportions for selected variables, a Chi-square or Fisher’s Exact test was used dependent on the number of cases in the tested categories. To account for multiple significance testing and the exploratory nature of this analysis, Bonferroni adjustments were made to reduce the significance level to <0.01. Free-text responses to the question relating to cannula malposition and dislodgement were manually categorised and coded into the reason leading to the adverse patient outcome.

## Results

### Centre characteristics and peripheral cannulation methods

A total of 391 individual responses were received among 45 countries from 911 distributed surveys; two respondents did not provide any clinical data and were excluded from analysis, yielding an overall response rate of 43%. The possibility of double enrolments in centres cannot be ruled out. Approximately half (56%) of the responses came from the Americas and one-quarter from Europe (28%); the greatest number received by country were from the United States (n = 181) and the United Kingdom (n = 32). Eighty-five percent of ECMO centres were in an academic teaching hospital and 43% had been providing ECMO for more than 15 years. The percentage of responses by world region and centre characteristics is shown in Table 1. Respondent centres included neonatal, paediatric and adult patient populations receiving ECMO for respiratory, cardiac and cardiopulmonary resuscitation reasons (Table 2).

**Table 1:** ECMO centre characteristics by world region.

Region	Respondent centres^˄^	Located in academic teaching hospital^#^	Years providing ECMO^¥^ (n, % within region)				
	(n, %)	(n, % within region)	0–2	>2–5	>5–10	>10–15	>15
Africa	3 (0.8)	1 (33)	0	2 (67)	1 (33)	0	0
Americas	215 (56)	179 (83)	15 (7.0)	29 (13)	45 (21)	15 (7.0)	110 (51)
Asia	37 (9.5)	27 (73)	6 (16)	12 (32)	15 (40)	2 (5)	2 (5)
Europe	109 (28)	101 (93)	1 (0.9)	10 (9.2)	40 (37)	15 (14)	42 (39)
Australia/New Zealand	20 (5.2)	20 (100)	0	3 (15)	5 (25)	2 (10)	10 (50)

^˄^N = 384

^**#**^N = 328

^¥^N = 382; Percentage figures may not add up to 100 due to rounding.

**Table 2:** Percentage of annual ECMO cases and reasons for ECMO by patient population.

	**Neonate**^**˄**^ **(n, %)**	**Paediatric**^**#**^ **(n, %)**	**Adult**^**¥**^ **(n, %)**
Cases per year*			
1–6	108 (46)	128 (49)	65 (22)
>6–12	50 (21)	64 (25)	37 (13)
>12–30	60 (25)	56 (21)	95 (32)
>30	18 (7.6)	12 (4.6)	97 (33)
	**Neonate (n, %)**	**Paediatric (n, %)**	**Adult (n, %)**
Reason for ECMO^~^			
Cardiac	205 (63)	235 (69)	265 (75)
Respiratory	201 (62)	224 (66)	279 (79)
CPR	126 (39)	159 (47)	181 (51)

Not all respondents provided responses to each question; Respondents by category

^˄^Neonate, N = 236

^#^Paediatric, N = 260

^¥^Adult, N = 294 (respondents reporting no cases per year of a particular patient population removed from denominator)

*Percentage figures may not add up to 100 due to rounding

^~^Items do not add to 100% as multiple options could be selected; CPR–cardiopulmonary resuscitation.

The most common peripheral cannulation method was percutaneous Seldinger technique for venovenous access and return (60% each) and venoarterial access (41%); and direct cut-down method for venoarterial return (43%). The most commonly reported cannulation vessels were jugular vein with double-lumen cannula for neonatal and paediatric venovenous ECMO (85% and 65% respectively); femoral-jugular vein (39%) for adult venovenous ECMO; jugular vein-carotid artery for neonate (96%) and paediatric (64%) venoarterial ECMO; and femoral vein-femoral artery (81%) for adult venoarterial ECMO.

### Infection prevention for peripheral cannulation, cannula dressing and circuit access

Hand hygiene was routinely performed by 89% of respondents, and 91% prepped the skin with antiseptic prior to peripheral cannula insertion. During peripheral cannulation, respondents indicated the use of maximal barrier precautions as follows: 90% used cap/hat, mask and sterile gown; 91% used sterile gloves; and 85% used a sterile full body drape. Three-quarters of respondents stated that a sterile occlusive dressing was used to cover the insertion site.

Hand hygiene was performed by 83% of respondent centres when replacing dressings. Clinical practice during cannula dressing changes was identified as: use of a cap/hat in almost half of respondents (47%), over two-thirds used a mask (68%); and just over three-quarters used sterile gloves (79%), while 13% used non-sterile gloves. Forty percent stated that an aseptic ‘no-touch’ technique was used during dressing replacement. Prior to accessing circuit ports, reported precautions undertaken included hand hygiene (77%), non-sterile glove (60%) and sterile glove (20%) use.

The preferred antiseptic was chlorhexidine gluconate (CHG) in alcohol for skin antisepsis prior to cannula insertion and during dressing changes (53% and 54% respectively). Isopropyl alcohol swab was most common for disinfection of circuit access ports (39%). Table 3 shows the top five preferred antiseptics used for cannula insertion and maintenance, and circuit port access.

**Table 3:** Preferred antiseptics for cannula insertion and maintenance (cutaneous application), and circuit access port.

Cannula insertion^˄^	(n, %)	Cannula maintenance (dressing) ^#^	(n, %)	Circuit access port^¥^	(n, %)
CHG–alcohol	186 (53)	CHG–alcohol	186 (54)	Isopropyl alcohol swab	130 (39)
Povidone iodine–alcohol	76 (22)	CHG, aqueous	78 (22)	CHG–alcohol	106 (32)
CHG, aqueous	66 (19)	Povidone iodine–alcohol	44 (13)	No antisepsis or no-touch technique used	34 (10)
Isopropyl alcohol 70%	10 (2.9)	No antiseptic	15 (4.3)	CHG, aqueous	33 (10)
Octenidine–propanol	4 (1.1)	Isopropyl alcohol 70%	9 (2.6)	Povidone iodine–alcohol	17 (5.1)

Not all respondents provided responses to each question

^˄^N = 348

^#^N = 347

^¥^N = 334; Percentage figures do not add up to 100 as only the top 5 are reported here; CHG–chlorhexidine gluconate.

### Peripheral cannula infection surveillance

Most centres monitored for cannula site infection by either routine visual inspection through the dressing or during dressing change (84%), swab or culture on signs of infection (31%) or palpation through the dressing (18%). Routine collection of site swabs or subcutaneous needle aspiration of suspicious cannula sites was infrequently performed.

### Peripheral cannula dressing and line securement

The frequency of routine dressing replacement was most commonly ‘as required’ (i.e. soiled or bloody) or every 1–3 days (both 29%), followed by daily (20%). No centres reported that dressings were left in situ for greater than seven days. Three-quarters of peripheral cannula sites were dressed with a transparent semi-permeable dressing–40% of these with a plain transparent dressing; 21% with an integrated (1-piece) CHG-impregnated dressing; and 15% with a CHG disk plus transparent dressing over the top. Gauze and tape dressing was reported by 16% of respondents. There was widespread uptake of CHG-containing products for cannula dressing with 28 countries reporting their use; the highest users of such products being the Americas (40% of peripheral cannula dressings contained CHG) compared with the lowest being Australia/New Zealand (21%).

Peripheral ECMO cannulas were almost always sutured at the insertion site with 93% of respondents reporting this practice. Comparing regional variations in suturing practice, the Americas (96%; 95% CI 92, 98) and Europe (96%; 95% CI 90, 99) reported the most frequent use of this securement method compared with Australia/New Zealand reporting the lowest (68%; 95% CI 46, 85; Chi-square 50.6; *p* <0.0001). When examining the securement method primarily used along the length of the ECMO line/circuit tubing to prevent decannulation or dislodgement, suturing directly to the skin (i.e. leg, neck) was the most prevalent method (45%) followed by securing to a bed or other fixed object (including use of taping, clipping or tubing holder device, 19%). Use of a sutureless securement device (SSD) or adhesive fabric tape/bandage was 16% and 10% respectively. Forty-eight percent routinely used two fixation points along femoral line while approximately one-quarter used more than two (26%) or one (21%) point of fixation. Table 4 describes regional variations in methods of peripheral ECMO line securement.

**Table 4:** Peripheral ECMO line securement practices by world region.

Region	Suturing practice at cannula insertion site (%)^˄^			Primary securement method along ECMO line/circuit tubing (%) (not all methods listed)					Fixation points along femoral (leg) ECMO line (%)^˄^		
	Always	Some-times	Never	Direct skin suture	Secure to bed/ other fixed object^#^	SSD	Adhesive bandage or tape	Suture plus other method^¥^	>2	2	1
**Africa**	67	33	0	100	0	0	0	0	67	33	0
**Americas**	96	3.6	0.5	50	21	14	7.3	3.6	30	50	21
**Asia**	80	6.4	13	63	6.4	13	16	3.2	45	55	0
**Europe**	96	3.0	1.0	37	20	17	12	9.1	14	53	32
**Australia/New Zealand**	68	0	32	16	16	42	16	0	21	47	31

^˄^Figures may not add up to 100% due to rounding

^#^Includes taping, clipping or use of tubing holder device, or combination of any of these

^¥^Other method could include any of the other primary securement methods listed; SSD–sutureless securement device (refers to commercial adhesive product).

Concerningly, one-third (34%) of respondents reported the occurrence of a cannula/line malposition, dislodgement or accidental decannulation leading to an adverse patient outcome at their centre in the last five years. Over half of the respondents to this question provided narrative data (61 respondents described 71 adverse events) from which the leading cause of malposition, dislodgement or decannulation was inadequate securement (Table 5).

**Table 5:** Causes of ECMO cannula/line malposition or dislodgement resulting in an adverse patient event.

Cause	Episodes^˄^ (n, %)
Inadequate/ineffective securement	20 (28)
During insertion or manipulation of cannula	10 (14)
Patient removed cannula	9 (13)
During turning or bathing patient	8 (11)
During transport of patient	3 (4.2)
Cannula material failure	2 (2.8)
During ambulation	1 (1.4)
Specific cause not stated	18 (25)

^˄^N = 71; Based on narrative accounts from 61 respondents.

Associations between cannula/line adverse events (malposition or dislodgement/decannulation) and bedside practices revealed that adult centres handling greater than 30 cases per year reported more adverse events than those performing 1–6 cases per year (56% [95% CI 46, 66] vs 22% [95% CI 13, 35]; Chi-square 16.17; *p* <0.001). There were no significant differences in ECMO volume per year and a cannula adverse event in neonates and paediatric centres. Counterintuitively, 34% (95% CI 30, 41) of centres which always sutured at the cannula insertion site reported adverse line events whereas, in contrast, centres which never sutured reported none (95% CI 0, 28), however this failed to meet the *a priori* statistical significance level (Chi-square 4.40; *p* = 0.04). Length of ECMO experience had no association with adverse line events with centres providing ECMO for 0–2 years reporting comparable cannula adverse event rates than centres with over 15 years of ECMO experience (17% [95% CI 5, 40] vs 36% [95% CI 29, 44; Fishers Exact test, 0.12; *p* = 0.116). Similarly, there were no significant associations between a cannula adverse event occurring and whether the ECMO centre was in an academic teaching hospital or not (Chi-square 2.61; *p* = 0.272).

### Circuit access

The number of times for daily circuit access was 0–5 for more than half (54%) the respondent centres. Access occurred to perform blood sampling for oxygenator blood gas analysis (93%) and three-quarters of respondents reported (76%) undertaking haemodialysis or haemofiltration via the circuit. Table 6 describes circuit access practices.

**Table 6:** Circuit access practices.

**Predominant team member for day-to-day circuit** **access (*N* = 332)**	**Frequency**	**%**^**˄**^
Nurse	131	39
Perfusionist	115	35
ECMO specialist^#^	37	11
Respiratory therapist	28	8.4
Physician or medical doctor	21	6.3
**Number of times circuit accessed per day (*N* = 335)**	**Frequency**	**%**^**˄**^
0–5	182	54
>5–10	50	15
>10–15	38	11
>15–20	20	5.9
>20	47	14
**Reason for accessing the circuit (*N* = 338)**	**Frequency**	**%**^**¥**^
Blood sampling for oxygenator blood gas analysis	316	93
Haemodialysis or haemofiltration	262	76
Blood sampling for patient blood test	139	41
Administering drug infusions	136	40
Administering blood products	134	40
Blood sampling for blood culture	123	36
Plasmapheresis or total plasma exchange	115	34
Administering drug or electrolyte bolus	94	28

^˄^Figures may not add up to 100% due to rounding

^#^Defined here as a specialty trained registered nurse or respiratory therapist—figure includes respondents (2/37) who stated both ‘ECMO specialist’ and ‘perfusionist’.

^¥^Items do not add to 100% as multiple options could be selected.

Circuit connection methods were reported most frequently as luer-lock for syringe (55%), infusion line (38%), and extracorporeal line (for haemodialysis/haemofiltration or plasmapheresis; 65%), whereas connections using a needleless valve were less frequent (syringe, 27%; infusion line, 18%; extracorporeal line, 8%). Up to 13% of respondents reported either connection method was used.

### Guidelines for ECMO line management practices

Over three-quarters (76%) of the respondents had a written policy or guideline for bedside ECMO line management while the remainder did not (22%) or were unsure (3%) if a policy existed at their centre. Most (78%) believed that an international evidence-based guideline for ECMO line management would be beneficial to inform or improve bedside practices.

## Discussion

Results from this survey provide insights into bedside management of peripheral ECMO lines among 45 countries with evidence of practice variation in infection precautions, dressings, securement and connections. A number of management aspects appear inconsistent with available recommendations including those from the ELSO Infectious Disease Task Force[22].

### Infection precautions and surveillance

The use of standardised infection prevention practices relevant to central venous catheters[25] appears to be commonplace during ECMO cannula insertion with the vast majority of centres reporting adherence to maximal barrier precautions, performing hand hygiene, disinfecting the patient’s skin with alcohol-based antiseptic, and applying a sterile full-body drape. Although hand hygiene had a high reported compliance for cannula insertion and dressing replacement, compliance was lower prior to circuit access with nearly one-in-four respondents reporting that hand hygiene was not routinely performed. This may reflect perceptions or attitudes that the cannulas represent a greater risk for microbial contamination than does entering the circuit ports. Should this be the case, it contravenes recommendations of the ELSO Infectious Disease Task Force[22] to treat ECMO circuits as protected central lines as both cannula handling and port accessing involve breaking the sterile circuit and potentially allow microbial contamination. However, behavioural reasons may influence hand hygiene practices such that compliance may be poorer depending on the clinical context (e.g. emergency situation), which we did not specifically survey.

Similarly, avoiding unnecessary circuit access is a recommended strategy to reduce the risk for infection[22]. Our results demonstrate that ECMO centres are adhering to this recommendation with no more than five access occasions per day for over half of the respondents. Conversely, over one-quarter of respondents reported administering intermittent drug or electrolyte boluses via the circuit whereas only the administration of continuous infusions is recommended to minimise breaking circuit sterility[22]. This may reflect a shortage of access sites in these critically ill patients but using the ECMO circuit as an intermittent vascular access port is against current practice recommendations and should not occur.

Numerous types of antiseptic preparations were reported for cannula insertion and maintenance, and for accessing the circuit. Alcohol-based CHG was most commonly reported as the preferred antiseptic for both cannula insertion and maintenance, whilst its use was slightly less than alcohol swabs for circuit access. Alcohol was also used to disinfect access ports by the majority of centres surveyed by Glater-Welt et al.[21]. This practice conflicts with ELSOs recommendation to use CHG preparations for circuit antisepsis rather than alcohol or povidone iodine[22]. It is unclear from ELSO whether CHG should be contained within an alcohol-based or aqueous preparation. The prevalent use of CHG in alcohol identified in this survey aligns with guidelines relevant to central lines[25–27]. This combined preparation has proven more effective than either CHG or alcohol used alone due to both immediate and sustained disinfecting action[28]. However, further data regarding skin antisepsis regimens including the influence of aqueous or alcohol-based solutions on infection outcomes is needed[29].

Aqueous-based CHG preparation was identified as one of the top five preferred line antisepsis agents in this survey. Its use appears to correspond with anecdotal evidence from centres whose local policy dictates alcohol avoidance due to the potential to cause damage to plastics used in ECMO cannulas and circuit componentry. However, there is little evidence to corroborate this. Past reports have implicated acetone contained within the solution as the destructive agent, not alcohol alone[30]. Investigation into the effects of alcohol in this context would clarify this practice discrepancy.

Respondent centres identified the most common circuit connection on ports or stopcocks is via a luer-lock (‘screw-in’) method whereas the recommendation is for the use of needleless valves which are considered more reliably decontaminated with disinfecting solutions[22]. Evidence regarding the effectiveness of needleless connectors in infection prevention is unclear. They appear to reduce microbial colonisation and catheter-related BSI, but the mechanical type needleless connectors may increase infection risk[25, 26]. Evidence is based on comparisons between needleless connectors and stopcocks or caps used for intravascular devices and does not account for extracorporeal circuit ports. Interestingly, a small number of centres reported using passive disinfection, usually containing alcohol, on circuit ports. Disinfection caps have been shown to reduce intraluminal contamination and central catheter-related BSI rates[31] and thus have potential as a practicable disinfection strategy prior to making connections to the ECMO line ports. Research on the use of needleless connectors and disinfection caps in the ECMO context is needed.

Visual cannula site inspection, followed by swab and culture, was the most commonly reported local infection surveillance method. Therefore, it is important that any dressing placed over the insertion site is transparent to allow for continual inspection. Data on ECMO CRI and colonisation is limited and mostly derived from single-centre cohorts[1, 17, 18, 32] but is nonetheless of increasing concern as evidence evolves. Most recently Allou et al.[1] found that CRIs were frequent in patients supported by peripheral ECMO with 17.7% of patients developing this complication. Importantly it is likely the magnitude of the problem is not fully appreciated: CRIs may be under-reported in the absence of a consensus definition[15] and thus this complication may be under-recognised in clinical practice and less reliably reported in the evidence base. Patients on ECMO generally have multiple invasive lines in addition to the large-bore ECMO lines which increases the number of entry portals for intraluminal contaminants and constitutes an increased risk for BSI among other factors[6, 33]. Routine changeout of ECMO lines is unfeasible due to the high risk of life-threatening complications and patient cardiorespiratory instability precludes this as an option when potential or proven infections occur. Careful adherence to preventive measures and vigilant patient monitoring is vital to avoid this eventuality. There is a clear need for further research as there are no guidelines for CRI diagnosis and management at present.

### Dressing and securement of peripheral ECMO lines

Dressing changes most commonly occurred either when necessary or between one to three days frequency, followed by daily. Glater-Welt et al.[21] similarly reported that ‘when necessary’ dressing changes were carried out by most of their surveyed centres. Transparent dressing changes for central catheters are recommended every seven days or sooner if dressing adherence or soiling is an issue[26]. The need for frequent dressing changes reported for ECMO patients may reflect cannulation site bleeding issues, however, dressing disruption is a major risk factor for catheter-related infections and should be avoided where possible[34]. The ELSO general guidelines[20] do not specify a frequency for cannula site antisepsis except to recommend that sites be cleaned ‘frequently’. The development of a cannula dressing regimen with replacement frequencies specific to ECMO is needed.

CHG-impregnated dressing products have been shown to reduce central catheter-related BSI and colonisation compared with standard transparent dressings[35], appear to be cost effective in adult intensive care patients[36, 37], and are recommended for use in patients over the age of two months[27]. Our finding that CHG dressing products, including disks and integrated gel dressings, are used by more than half the countries surveyed suggests uptake of this evidence in ECMO practice. Use of an integrated transparent CHG dressing product is more practical in ECMO because, unlike disks, this dressing type allows visualisation of the cannula site and eliminates the need for two products. Data on clinical efficacy at reducing infection as well as safety and cost-effectiveness is needed to justify the routine use of CHG dressings in ECMO.

In compliance with ELSO recommendations, the securement of peripheral cannulas to the skin in at least two locations to prevent inadvertent decannulation appears common practice[20], as does suturing at cannulation sites, yet the concerning reports of adverse patient events associated with cannula dislodgement highlights the need to clearly define optimal line securement. We identified inadequate securement as the top cause of adverse patient outcomes resulting from cannula malposition or dislodgement. Many of the other stated reasons (i.e. during ambulation, transport and patient cares) may also be attributed to suboptimal dressing and securement techniques. Maintaining line security throughout the duration of ECMO is an absolute patient safety priority to avoid decannulation-related life-threatening emergencies[20]. The present trend towards less sedation and early mobilisation in certain patient groups[38] further underlines this imperative. Our respondents reported numerous securement methods likely indicative of the heterogeneity of patient populations, cannulation methods, circuit configurations, and models of care practiced. Methods included various commercial products (i.e. ‘Grip-Lok’ style devices, Foley catheter devices, colostomy ring patches) not designed and/or marketed specifically for ECMO use. Some methods may be risky if used in novel ways outside of manufacturer recommendations[39]. Rigorous testing of available products or the development of ECMO-specific devices would be beneficial to ensure they allow for safe and robust fixation of large ECMO lines.

Cannula adverse events were higher in high-volume adult centres and no difference was identified in adverse events rates between centres with less than two years of ECMO provision and those with over 15 years. Reports of more cannula malposition or dislodgement in centres which always suture at the cannula insertion site compared with sites who never suture did not meet the *a priori* statistical significance level but is of clinical significance. The finding is somewhat counterintuitive as suturing would be expected to enhance securement of the ECMO cannula. This may indicate greater vigilance toward line monitoring and management in centres that do not routinely suture cannulas because this practice is thought to present a greater risk for the occurrence of adverse events. It must be noted though that reporting and recall bias may have affected the reporting of these adverse events.

### Clinical practice guidelines for ECMO lines

There is presently a lack of high-quality evidence to guide ECMO line management practices. Recommendations from the ELSO[20, 22] are largely derived from consensus guidelines applicable to smaller intravascular devices[25, 26] and lack ECMO-specific data. Therefore, currently available evidence may be insufficient for the larger specialised lines used in ECMO. This is supported by the belief of most ECMO respondents that an international evidence-based guideline is needed to inform bedside practices in this regard.

### Limitations

Due to the cross-sectional design our findings may not represent all practices in the ECMO community. Similarly, participants from the United States contributed almost half of the responses but this was expected given that the largest number of ECMO centres is present in this location. However, our survey had a good geographic distribution with responses from 45 of the 51 countries surveyed. Findings may also be limited by issues inherent with self-reported data including recall bias; social desirability bias; and question interpretation by respondents whose first language is not English. Our response rate is based on individual respondent numbers. It was not possible to calculate a per centre rate as we chose not to identify participant or institutional details, or record IP addresses for anonymity. Therefore, despite explicit instructions requesting one participant per centre, it is evident that some double enrolments occurred in this survey. We chose to focus on the pragmatic, routine, hands-on line practices undertaken at the bedside; in the interests of length our survey did not cover in-depth all line management aspects or issues (i.e. central cannulas, cannula bleeding, surveillance cultures, antimicrobial use, and infection rates), some of which have been previously surveyed[21, 40]. Nonetheless, our study is the first to comprehensively examine line dressing, securement and access practices to our knowledge.

## Conclusions

Variable practices regarding ECMO line management exist worldwide based on recommendations lacking specific ECMO content and context. This survey identified evidence gaps regarding several aspects of patient management, specifically antiseptic agents, dressing products and regimens, securement methods and needleless valves. The vulnerability of ECMO patients for significant morbidity and mortality associated with line-related complications and ECMOs expanding use worldwide warrants targeted research to develop best practice guidelines for clinicians managing ECMO lines.

## Supporting information

S1 Appendix Questionnaire tool. ECMO line bedside practices.
(PDF)

S1 Dataset Minimal anonymized data set. (XLSX)
