# Supplementary material for: Cannula and circuit management in peripheral extracorporeal membrane oxygenation: An international survey of 45 countries
Source: PLoS One. 2019 Dec 30;14(12):e0227248. doi: 10.1371/journal.pone.0227248 (PMC6936833; doi:10.1371/journal.pone.0227248)
Supplement: S1 Appendix — ECMO line bedside practices. (PDF) [file pone.0227248.s001.pdf]

ECMO line bedside practices
Survey instructions

Dear ECMO colleague

ECMO line-associated complications can have serious patient consequences. Vigilant bedside practices are important to prevent circuit or cannula-related infection, and cannula migration or accidental decannulation.

This survey is being conducted to identify current peripheral ECMO line (cannula and circuit) bedside practices worldwide regarding infection precautions, securement, dressings and circuit access. Knowledge of these practical aspects of care will help identify any gaps for further research to support the development of evidence-based guidelines which are currently lacking in this area.

We greatly appreciate your completion of our questionnaire by **one ECMO director or coordinator with knowledge of your specific day-to-day peripheral ECMO line practices** on behalf of your ECMO centre/service. **Please select the response(s) which best describes standard routine practice.**

Your voluntary survey participation and submission verifies your consent to participate. Your responses are anonymous and you won't be asked to disclose identifying information. It should take around 10 minutes to complete.

Thank you for your time to support continuous quality improvement in ECMO.

Please contact us if you need further information.

Kind regards
[contact details]

Endorsed by the International ECMO Network (ECMONet)

Demographics

Questions 1 to 11 refer to your current work environment and ECMO methods.

1. Which country is your ECMO centre / service located in?

2. Is your ECMO centre / service located within an academic teaching hospital?

- Yes
- No
- Unsure

3. How long has your centre / service been providing ECMO?

- ◯ 0-2 years
- ◯ >2-5 years
- ◯ >5-10 years
- ◯ >10-15 years
- ◯ More than 15 years

4. Which professionals are core members of your ECMO team? (choose all that apply)

- ☐ Nurses
- ☐ Perfusionists
- ☐ Respiratory therapists
- ☐ Physicians / medical doctors
- ☐ Cardiothoracic surgeons
- ☐ Other (please specify)

5. For each ECMO population below, approximately how many patients are placed on ECMO each year?

	0	1-6	>6-12	>12-30	More than 30
a) Neonate	◯	◯	◯	◯	◯
b) Paediatric	◯	◯	◯	◯	◯
c) Adult	◯	◯	◯	◯	◯

6. For what reasons is ECMO support provided? (choose all that apply)

	Not applicable	Cardiac	Respiratory	Cardiopulmonary resuscitation (CPR)
a) Neonate	☐	☐	☐	☐
b) Paediatric	☐	☐	☐	☐
c) Adult	☐	☐	☐	☐

7. Does your ECMO centre / service have a written policy or guideline (or statement within a policy / guideline) covering bedside ECMO line management?

- ◯ Yes
- ◯ No
- ◯ Unsure

8. What is the most frequently performed cannula insertion method for peripheral veno-venous ECMO?

	**Direct cut-down/surgical**	**Seldinger technique** (percutaneous vessel puncture, guidewire placement and serial dilation)	**Semi-seldinger** (combination of cut-down exposure and Seldinger technique)	**Not applicable**
a) Access (drainage) cannula	○	○	○	○
b) Return cannula	○	○	○	○

Other (please specify)

9. What is the most frequently performed cannula insertion method for peripheral veno-arterial ECMO?

	**Direct cut-down/surgical**	**Seldinger technique** (percutaneous vessel puncture, guidewire placement and serial dilation)	**Semi-seldinger** (combination of cut-down exposure and Seldinger technique)	**Not applicable**
a) Access (drainage) cannula	○	○	○	○
b) Return cannula	○	○	○	○

Other (please specify)

10. What is the most frequently used peripheral access (drainage) and return cannula placement for veno-venous ECMO?

	Not applicable	Femoral vein-Femoral vein	Femoral vein-Jugular vein	Jugular vein-Femoral vein	Jugular vein with double lumen cannula
a) Neonate	○	○	○	○	○
b) Paediatric	○	○	○	○	○
c) Adult	○	○	○	○	○

Other (please specify)

11. What is the most frequently used peripheral access (drainage) and return cannula placement for veno-arterial ECMO?

	Not applicable	Femoral vein-Femoral artery	Femoral vein-Axillary artery	Jugular vein-Carotid artery	Jugular vein-Axillary artery	Jugular vein-Femoral artery
a) Neonate	○	○	○	○	○	○
b) Paediatric	○	○	○	○	○	○
c) Adult	○	○	○	○	○	○

Other (please specify)

Cannula infection precautions and dressings

Questions 12 to 18 refer to infection precautions undertaken for insertion and ongoing cannula management at the bedside. Choose the answer which best describes standard practice for the **majority of ECMO patients with peripheral cannulation**

12. Which aseptic techniques and barrier precautions are routinely used during peripheral cannula insertion? (choose all that apply)

- ☐ Hat/cap
- ☐ Mask
- ☐ Hand washing (water and soap or alcohol-based hand rub)
- ☐ Sterile gown
- ☐ Sterile gloves
- ☐ Sterile full body drapes
- ☐ Skin preparation with antiseptic
- ☐ Sterile occlusive dressing to cover insertion site
- ☐ Other (please specify)

13. What is the preferred skin antiseptic agent for peripheral cannula insertion?

- ◯ Chlorhexidine gluconate in alcohol
- ◯ Chlorhexidine gluconate aqueous (non-alcoholic)
- ◯ Povidone iodine in alcohol
- ◯ Isopropyl alcohol 70%
- ◯ Other (please specify)

14. How often are peripheral cannula site dressings replaced as part of routine care?

- ◯ Daily
- ◯ 1-3 days
- ◯ >3-5 days
- ◯ >5-7 days
- ◯ More than 7 days
- ◯ Only as needed (soiled, bloody)

15. Which aseptic techniques and barrier precautions are routinely used during dressing replacement? (choose all that apply)

- ☐ Hat/cap
- ☐ Mask
- ☐ Hand washing (water and soap or alcohol-based hand rub)
- ☐ Sterile gown
- ☐ Sterile gloves
- ☐ Non-sterile gloves
- ☐ Aseptic "non-touch" technique
- ☐ Other (please specify)

16. What is the preferred antiseptic agent for decontaminating the cannula site during routine dressing replacement?

- ◯ Chlorhexidine gluconate in alcohol
- ◯ Chlorhexidine gluconate aqueous (non-alcoholic)
- ◯ Povidone iodine in alcohol
- ◯ Isopropyl alcohol 70%
- ◯ Not applicable (no antiseptic is used)
- ◯ Other (please specify)

17. What is the preferred sterile dressing used to cover peripheral cannula insertion sites?

- ◯ Gauze and tape
- ◯ Transparent semi-permeable dressing
- ◯ 1-piece transparent chlorhexidine gluconate-impregnated dressing ("Tegaderm CHG")
- ◯ 2-piece chlorhexidine gluconate-impregnated disk/sponge ("Biopatch") with transparent dressing over top
- ◯ Silver-impregnated dressing
- ◯ Other (please specify)

18. What surveillance methods are used to detect local infection at the cannulation site? (choose all that apply)

- ☐ Routine palpation through an intact dressing
- ☐ Routine visual inspection when changing the dressing
- ☐ Routine insertion site swab and culture
- ☐ Insertion site swab and culture only on suspicion of infection
- ☐ Subcutaneous needle aspiration on suspicion of infection
- ☐ Other (please specify)

Cannula / line securement

Questions 19 to 22 refer to securement of peripheral ECMO lines. Choose the answer which best describes standard practice for the **majority of peripheral ECMO patients**.

19. Following peripheral cannulation, is the cannula sutured at the insertion site?

- ◯ Always
- ◯ Sometimes
- ◯ Never

20. Securement along the length of the ECMO line or circuit tubing to prevent accidental decannulation / dislodgement is achieved primarily by:

- ◯ Suturing directly to skin (eg. thigh, leg, neck)
- ◯ Commercial sutureless adhesive fixation device
- ◯ Fabric adhesive bandage or tape
- ◯ Clipping or taping to the bed or other fixed object
- ◯ Other method (please specify)

21. Excluding the insertion site, how many fixation points are routinely used along the length of each femoral ECMO line for securement?

- ◯ 1 fixation point
- ◯ 2 fixation points
- ◯ More than 2 fixation points
- ◯ Not applicable (femoral cannulations are not performed)

22. Has there been an ECMO cannula / line malposition, dislodgement, or accidental decannulation that has resulted in an adverse patient event or outcome at your centre in the last 5 years?

- ◯ Yes
- ◯ No
- ◯ Unsure

If yes, please provide details (optional)

Circuit access

Questions 23 to 30 refer to circuit access practices at the bedside. Choose the answer which best describes standard practice for the **majority of ECMO patients**.

23. For what reason(s) is the ECMO circuit accessed (excluding pressure monitoring)? (choose all that apply)

- [ ] Blood sampling for oxygenator blood gas analysis
- [ ] Blood sampling for patient blood test / analysis
- [ ] Blood sampling for blood culture
- [ ] Administering drug infusions
- [ ] Administering drug or electrolyte boluses
- [ ] Administering blood products
- [ ] Haemodialysis or haemofiltration
- [ ] Plasmapheresis / total plasma exchange (TPE)
- [ ] Other (please specify)

24. Who accesses the circuit as part of their ECMO care responsibilities? (choose all that apply)

- [ ] Nurse
- [ ] Respiratory therapist
- [ ] Perfusionist
- [ ] Physician / medical doctor
- [ ] Other (please specify)

25. Who predominantly accesses the ECMO circuit on a day-to-day basis?

- ○ Nurse
- ○ Respiratory therapist
- ○ Perfusionist
- ○ Physician / medical doctor
- ○ Other (please specify)

26. What is the average number of times the ECMO circuit is accessed for an ECMO patient each day?

- ○ 0-5
- ○ >5-10
- ○ >10-15
- ○ >15-20
- ○ More than 20

27. Which hand hygiene or antisepsis measures are performed before accessing the circuit? (choose all that apply)

- ☐ Hand washing (water and soap or alcohol-based hand rub)
- ☐ Sterile gloves
- ☐ Non-sterile gloves
- ☐ Other (please specify)

28. Which type of antiseptic or technique is most commonly used to clean circuit ports / hubs before accessing the circuit?

- ○ Chlorhexidine gluconate in alcohol
- ○ Chlorhexidine gluconate aqueous (non-alcoholic)
- ○ Povidone iodine in alcohol
- ○ Isopropyl alcohol swab
- ○ "Non-touch" technique
- ○ Other (please specify)

29. Which method best describes connections made to the ECMO circuit for the following?

	**Needle-less (one-way valve) device**	**Luer-lock (direct screw-in)**	Either **needle-less valve** *or* **luer-lock**	Not applicable
a) Blood sampling or drug bolus syringe	○	○	○	○
b) Drug infusion line	○	○	○	○
c) Haemodialysis, haemofiltration or TPE line	○	○	○	○

30. Do you think a standardised, international evidence-based guideline for ECMO line management would be beneficial to inform or improve bedside practices?

- ◯ Yes
- ◯ No
- ◯ Unsure
